# Down‐regulation of miR‐26b induces cisplatin resistance in nasopharyngeal carcinoma by repressing JAG1

**DOI:** 10.1002/2211-5463.12135

**Published:** 2016-10-24

**Authors:** Lei Shi, Wei Yin, Zhiyu Zhang, Guanggang Shi

**Affiliations:** ^1^ Department of Otolaryngology‐Head and Neck SurgeryShandong Provincial Hospital Affiliated to Shandong UniversityJinanChina; ^2^ Department of Radiation OncologyHangzhou Cancer HospitalChina

**Keywords:** cisplatin resistance, FOXD3, JAG1, miR‐26b, nasopharyngeal carcinoma

## Abstract

Therapy against nasopharyngeal carcinoma (NPC) is hurdled by chemoresistance. Recent studies found that microRNA (miRNA) are important regulators of cancer resistance. In this study, we aimed to explore the role and mechanism of miR‐26b in regulating NPC cisplatin (CDDP) resistance. Real‐time PCR was used to evaluate miR‐26b levels in CDDP‐resistant and CDDP‐sensitive NPC cells, as well as human NPC tissues. MiR‐26b was ectopically overexpressed in CDDP‐resistant cells, followed by monitoring changes in cell viability and apoptosis. Interaction between JAG1 and miR‐26b was characterized by dual‐luciferase reporter assay. Furthermore, we investigated whether ectopic JAG1 expression reversed CDDP sensitivity induced by miR‐26b overexpression. The effect of FOXD3 down‐regulation on miR‐26b was also evaluated. Our results indicate that miR‐26b was lower in the CDDP‐resistant NPC cells, human NPC tissue, particularly in secondary metastases. Ectopic expression of miR‐26b sensitized NPC cells to CDDP. JAG1 is a target of miR‐26b, and its expression is inversely correlated with miR‐26b. Overexpression of JAG1 reversed the CDDP sensitivity induced by miR‐26b overexpression. FOXD3 expression was also down‐regulated in CDDP‐resistant NPC. FOXD3 promoted miR‐26b expression and down‐regulation of FOXD3 suppressed miR‐26b expression. Down‐regulation of miR‐26b is closely correlated with the CDDP resistance in NPC.

**Abbreviations**
AAV: adeno‐associated virus
CDDP: cisplatin
EBV: Epstein–Barr virus
EMT: epithelial mesenchymal transition
Fox: forkhead box
HEK: human embryonic kidney
NPC: nasopharyngeal carcinoma

Nasopharyngeal carcinoma (NPC) is a squamous cell carcinoma commonly found around the ostinum of the eustachian tube in the lateral wall of the nasopharynx. NPC is the most common type of cancer in head and neck region, and is a prevalent disease in southern China and Southeastern Asia 1. Although locoregional NPC is mostly curable, the majority of NPC is diagnosed at an advanced stage and metastatic NPC is responsible for the poor prognosis of patients. Currently, cisplatin (CDDP) is the first‐line treatment for NPC. However, high‐stage NPCs are not sensitive to CDDP and 82% of metastatic NPCs eventually relapse 2. According to the 7th edition of AJCC Cancer Staging Manual, the 5‐year survival rates of patients with advanced‐stage NPC are very low (62% for stage III and 38% for stage IV). Thus, there is an urgent need to elucidate the underlying mechanism of CDDP resistance, and find ways to sensitize NPCs to CDDP treatment.

MicroRNA are a class of noncoding RNA with the length of 18–25 nucleotides. MiRNA regulate protein expression post‐transcriptionally by targeting the 3′ UTRs of target mRNA, resulting in inhibited translation or the degradation of the targeted mRNA 3. Growing evidence indicates that the dysregulation of miRNA contributes to a number of cancer‐related events, such as proliferation, apoptosis, cell migration, and invasion 4. Thus far, a number of miRNA have been demonstrated to play an important role in NPC, including miR‐31 5, miR‐29c 6, miR‐138 7, etc. Recently, miR‐26b were found to be down‐regulated in colorectal cancer 8 and breast cancer 9, and miR‐26b is closely associated with the apoptosis and metastasis of cancer cells. The other member of miR‐26 family, miR‐26a, was also down‐regulated in NPC 10. Therefore, it is likely that miR‐26b is an important regulator of NPC. Understanding the underlying mechanism of miR‐26b in NPC may shed light on how NPC develops resistance to CDDP.

JAG1 is an oncogene that promotes metastasis and chemoresistance of a number of cancers through Notch signaling 11. High JAG1 expression is closely associated with cancer progression and poor patient survival. Recent studies found that JAG1 is regulated by miRNA, through which cells gain chemoresistance. A link between miR‐21 family and JAG1 was implicated previously in the study of autoimmune diseases 12. In NPC, the interaction between miR‐26b and JAG1 remains to be clarified.

The forkhead box (Fox) genes are evolutionarily conserved transcriptional factors that regulate a wide spectrum of biological processes. It is composed of 17 subfamilies (A to R), among which the FOXD3 is known to play a crucial role in carcinogenesis 13. FOXC2 was previously found to enhance chemoresistance in NPC via promoting epithelial mesenchymal transition (EMT) 14. FOXD3 was shown to regulate miR‐137 expression, through which FOXD3 regulates tumor growth and metastasis in human hepatocellular carcinoma 15. However, no studies have yet characterized the role of FOXD3 in NPC.

Herein, we sought to investigate the exact function of miR‐26a in NPC, with particular interest in its regulatory role of CDDP resistance. Here, we show that miR‐26b is prominently down‐regulated in CDDP‐resistant NPC compared to the CDDP‐sensitive NPC. On the other hand, ectopic miR‐26b expression was able to sensitize NPC to CDDP treatment. We also found that JAG1 is a target of miR‐26b. FOXD3 is also down‐regulated in NPC, and it regulates miR‐26b expression through binding to the promoter region of miR‐26b.

## Materials and methods

### Cell culture

Nasopharyngeal cancer cell lines CNE2, HNE1, and the CDDP‐resistant HNE1 (HNE1/DDP) were purchased from the Central Laboratory of Xiangya School of Medicine, Central South University. CNE2/DDP cell lines were kindly provided by the Department of Hematology, Zhujiang Hospital, Southern Medical University. Cells were cultured in RPMI‐1640 medium (Invitrogen, San Diego, CA, USA) supplemented with 10% heat‐inactivated FBS (Invitrogen, Pleasanton, CA, USA), 2 mm l‐glutamine, 100 U·mL^−1^ penicillin, 100 μg·mL^−1^ streptomycin in a humidified incubator at 37 °C, and 5% CO_2_. Besides, CDDP‐resistant HME/DDP and CNE2/DDP cells were maintained in RPMI‐1640 10% FBS medium containing 1 μg·mL^−1^ CDDP to maintain resistance.

### Cell viability assay

The cell viability after its response to CDDP in different conditions, was evaluated with Cell Counting Kit‐8 (CCK‐8) according to manufacturer's recommendations. The concentrations of CDDP were 0.625–20 μm, which was based on the peak CDDP concentration in plasma after administration of standard dosage of CDDP in patients 16. These concentrations of CDDP have also been used in other studies on the effects of CDDP in cancers 17. Briefly, 10 μL of CCK‐8 solution was added to cells cultured on a 96‐well plate, and the cells were incubated for 1 h at 37 °C. The absorbance at 450 nm of each well was measured on a microplate reader (Multiskan, Thermo Fisher Scientific, Waltham, MA, USA), which correlated with the viable cell number. Cell viability was expressed as:

$$\text{Cell viability}=\frac{\text{OD}\text{(experiment)}-\text{OD}\text{(blank)}}{\text{OD}\text{(control)}-\text{OD}\text{(blank)}}.$$

### Real‐time PCR

Total RNA from cells and tissues were isolated using the Total RNA Isolation System (Promega, Madison, WI, USA). For RT‐PCR analysis of miR‐26b, RNA samples were acquired using the mirVana miRNA Isolation kit (Ambion, Waltham, MA, USA). cDNA was synthesized from 1 μg total RNA using cDNA synthesis kit. RT‐PCR was performed using a thermal cycler (Eppendorf, Hamburg, Germany) and the DNA Master SYBR Green 1 kit (Roche Applied Sciences, Penzberg, Germany). The following primers were used in our study: 5′‐CGCCCTGTTCTCCATTACTT‐3′ (sense) and 5′‐CCAGTGCAGGGTCCGAGGT‐3′ (antisense) for miR‐26b; 5′‐AGTCACTGGCACGGTTGTAG‐3′ (sense) and 5′‐TCGCTGTATCTGTCCACCTG‐3′ (antisense) for JAG1; 5′‐AATAAGGATCCGCCGCCACCATGACCCTGTCTGGAGGCA‐3′ (sense) and 5′‐GCCGGTCTAGATCATTGAGAAGGCCATTTCGATAACGCTG‐3′ (antisense) for FOXD3; 5′‐TGCGGGTGCTCGCTTCGCAGC‐3′ (sense) and 5′‐CCAGTGCAGGGTCCGAGGT‐3′ (antisense) for U6. The levels of U6 were used as an internal control. Quantitations of RNA levels were performed using the 2^−ΔΔCt^ method.

### Western blot analysis

The RIPA lysis and extraction buffer (Thermo Fisher Scientific) was used to lyse cells. Lysates were centrifuged at 1000 ***g*** for 5 min at 4 °C. Concentration of the lysates was measured with bicinchoninic acid assay (Thermo Fisher). In electrophoresis, 30 μg lysates were loaded into each lane of 12% precast gels (Biorad, Hercules, CA, USA), and separated proteins were transferred on a polyvinylidene difluoride membrane (Invitrogen, Pleasanton, CA, USA). All antibodies were purchased from Abcam (Cambridge, MA, USA).

### Lentivirus packaging and gene transfection

To establish NPC cells stably expressing miR‐26b, JAG1 and FOXD3, we used a lentivirus‐based transfection system. Recombinant lentiviruses were produced by cotransfecting human embryonic kidney (HEK) 293T cells with the lentivirus expressing plasmids containing pri‐miR‐26b, JAG1 cDNA, or FOXD3 cDNA, and packaging plasmid (pMDLg/pRRE and pRSV‐Rev) using Fugene6 as a transfection reagent. Infectious lentiviruses were harvested 48 h after transfection. Cell debris in medium was removed by centrifugation and filtration through 0.45‐μm filters (Millipore, Billerica, MA, USA). NPC cells were transduced with the lentiviruses.

### Dual‐luciferase reporter assay

The 3′ UTR sequence of wild‐type or mutant JAG1 was amplified with PCR and cloned into the pGL3 vector (Promega). These 3′‐UTR vectors and the control vector pRL‐TK (Promega) coding for Renilla luciferase were cotransfected with miR‐26b mimics or negative control into HNE1 and CNE2 cells using Lipofectamine 2000 (Invitrogen, Pleasanton, CA, USA). At 48 h post transfection, the luciferase activity was measured using the Dual‐Luciferase Reporter Assay System (Promega). The luciferase values of Renilla were used to normalize firefly luciferase activities, and the data were presented as the ratios of firefly/Renilla values. The experiments were performed in triplicates.

### Clinical samples

Nasopharyngeal carcinoma samples, including primary and secondary tumors, were collected at the time of surgery from previously untreated patients. Normal tissues were collected as adjacent nonmalignant tissues. All samples were collected in Shandong Provincial Hospital Affiliated to Shandong University. Samples were snap‐frozen and stored at −80 °C. Approval to collect specimens was granted by Shandong Provincial Hospital Affiliated to Shandong University. Specimens were processed using procedures approved by the Shandong Provincial Hospital Affiliated to Shandong University.

### Statistical analysis

To evaluate the significance of differences, one‐ or two‐way ANOVAs were used followed by a Tukey's post hoc test. The relationship between two factors was determined by Spearman's correlation analysis. Differences were regarded statistically significant if *P* < 0.05.

## Results

### Down‐regulation of miR‐26b in CDDP‐resistant NPC cells and recurrent tumors

**Figure 1:**
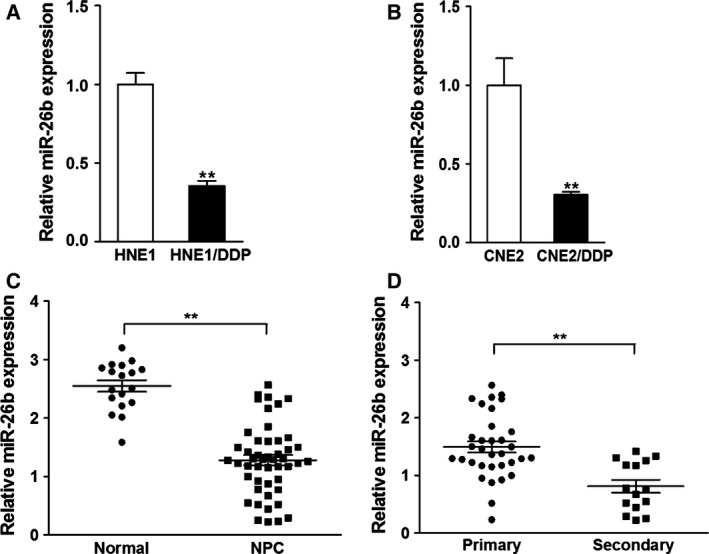
miR‐26b is down‐regulated in CDDP‐resistant NPC cells and recurrent tumors. (A, B) Relative miR‐26b expression levels of paired NPC CDDP‐resistant or CDDP‐sensitive cells were analyzed by qRT‐PCR. U6 RNA levels were used as internal control. Data were presented as mean ± SD from three independent experiments with triple replicates per experiment. **P < 0.01. (C) Relative miR‐26b expression levels in normal (*n* = 18) and NPC tissues (*n* = 48). **P < 0.01. (D) Relative expression levels of miR‐26b in primary (*n* = 33) and secondary NPC tumors (*n* = 15). **P < 0.01.

To demonstrate that down‐regulation of miR‐26b is an indicator of CDDP‐resistant NPC, the expression of miR‐26b was evaluated in the CDDP‐sensitive and CDDP‐resistant HME1 and CNE2 NPC cell lines, as well as NPC primary and secondary tumors. As shown in Fig. 1A,B, miR‐26b down‐regulation was seen in the CDDP‐resistant HNE1 and CNE2 cells. miR‐26b was also down‐regulated in NPC tissue compared to normal tissue (Fig. 1C). Notably, in secondary NPC, miR‐26b expression was also lower than primary tissue (Fig. 1D), indicating that NPC recurrence is associated with miR‐26b down‐regulation.

### MiR‐26b overexpression sensitized NPC to CDDP

**Figure 2:**
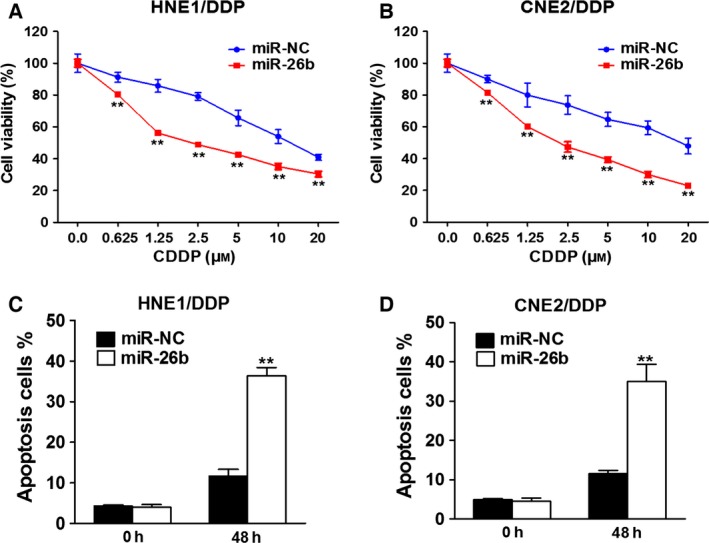
miR‐26b overexpression inhibits the drug resistance to CDDP in NPC. (A, B) HNE1/DDP and CNE2/DDP cells stably expressing miR‐NC or miR‐26b were treated with different concentrations of CDDP for 48 h, and analyzed by CCK‐8 assay. **P < 0.01. (C, D) HNE1/DDP and CNE2/DDP cells stably expressing miR‐NC or miR‐26b were treated with 10 μm CDDP for 0 or 48 h and cell apoptosis was analyzed by flow cytometry. **P < 0.01.

Since miR‐26b down‐regulation is a signature of CDDP‐resistant NPC, we reasoned that miR‐26b overexpression in CDDP‐resistant NPC cells might sensitize those cells to CDDP. First, we verified that overexpression of miR‐26b resulted in an up‐regulation of miR‐26b in both HNE1/DDP and CNE2/DDP cells (Fig. S1). Then, we showed that compared to cells receiving noncoding miRNA (miR‐NC), both HNE1 and CNE2 cells that were transfected with miR‐26b expression vector exhibited decreased cell viability in increasing concentrations of CDDP (0.625 μm to 20 μm;* P* < 0.01 for all concentrations) (Fig. 2A,B). Based on the cell viability analysis, the IC50 of cells transfected with miR‐NC was 12.943 μm and the IC50 of cells transfected with miR‐26b was 7.764 μm (Fig. 2A), while IC50 of cells transfected with miR‐NC was 16.376 μm and the IC50 of cells transfected with miR‐26b was 6.577 μm (Fig. 2B). Higher apoptotic rates were also seen in cells under miR‐26b overexpression (Fig. 2C,D).

### MiR‐26b regulates NPC chemoresistance by inhibiting JAG1

**Figure 3:**
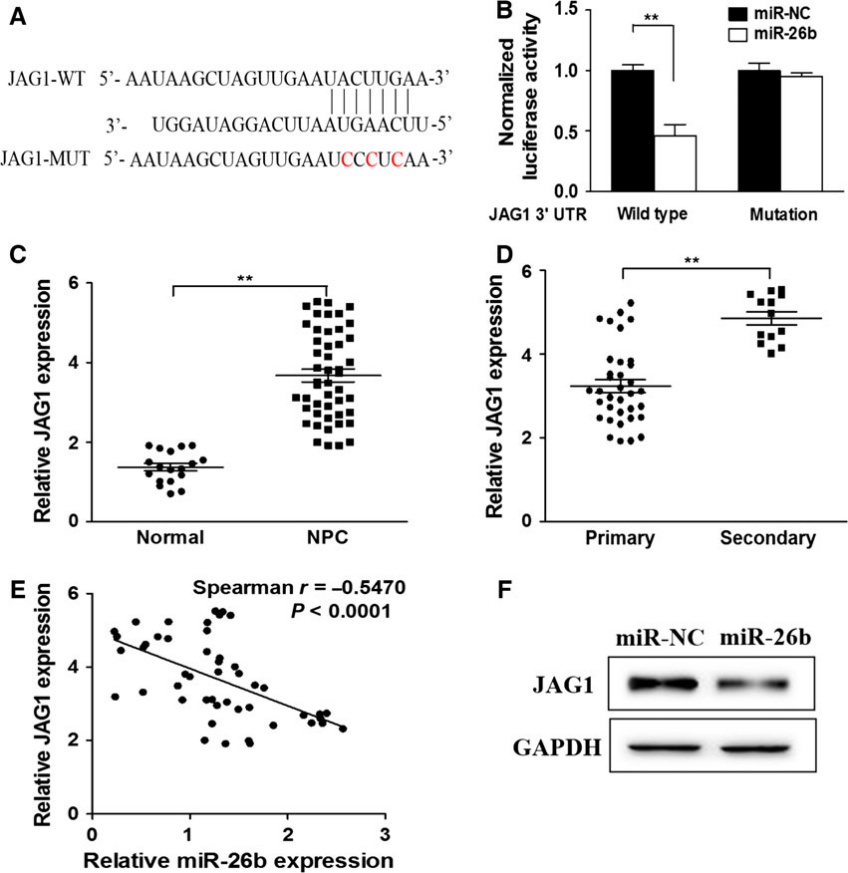
miR‐26b directly targets and inhibits oncogene CPD JAG1. (A) Putative seed‐matching sites (in bold and italic) or mutant sites (red) between miR‐26b and 3′‐UTR of JAG1. (B) Luciferase reporter assay was performed on HNE1 to detect the relative luciferase activities of WT and mutant JAG1 reporters. Renilla luciferase vector was used as an internal control. **P < 0.01. (C) Relative JAG1 expression levels in normal (*n* = 18) and NPC tissues (*n* = 48). GAPDH RNA levels were used internal control. **P < 0.01. (D) Relative expression levels of JAG1 in primary (*n* = 33) and secondary NPC tumors (*n* = 15). **P < 0.01. (D) Spearman's correlation analysis was used to determine the correlation between the expression levels of JAG1 and miR‐26b in human NPC specimens. (E) Total proteins of miR‐26b and miR‐NC‐expressing cells were subjected to western blot and detected for JAG1 expression levels.

To understand how miR‐26b exerts its tumor‐suppressive function, we used targetscan Release 7.1 18 to screen for genes that are post‐transcriptionally regulated by miR‐26b. It was found that JAG1 possesses a targeting sequence for miR‐26b at 3′ UT of the gene. Nucleotide mismatches between miR‐26b and JAG1 were introduced in these binding regions of JAG1 through mutation (Fig. 3A). Dual‐luciferase reporter assay indicated that miR‐26b negatively interacted with 3′ UTR of the wild‐type JAG1, as seen from the decreased luciferase activity after miR‐26b overexpression. In contrast, no interaction was seen between miR‐26b and mutant JAG1 (Fig. 3B). This evidence confirmed that JAG1 is directly inhibited by miR‐26b. In line with this, JAG1 was overexpressed in NPC tissue, particularly secondary tumors (Fig. 3C, D). Spearman's correlation analysis indicated a negative correlation between miR‐26b and JAG1 (Fig. 3E). Additionally, western blot analysis indicated that cells that received miR‐26b overexpression demonstrated a decreased level of JAG1 (Fig. 3F).

**Figure 4:**
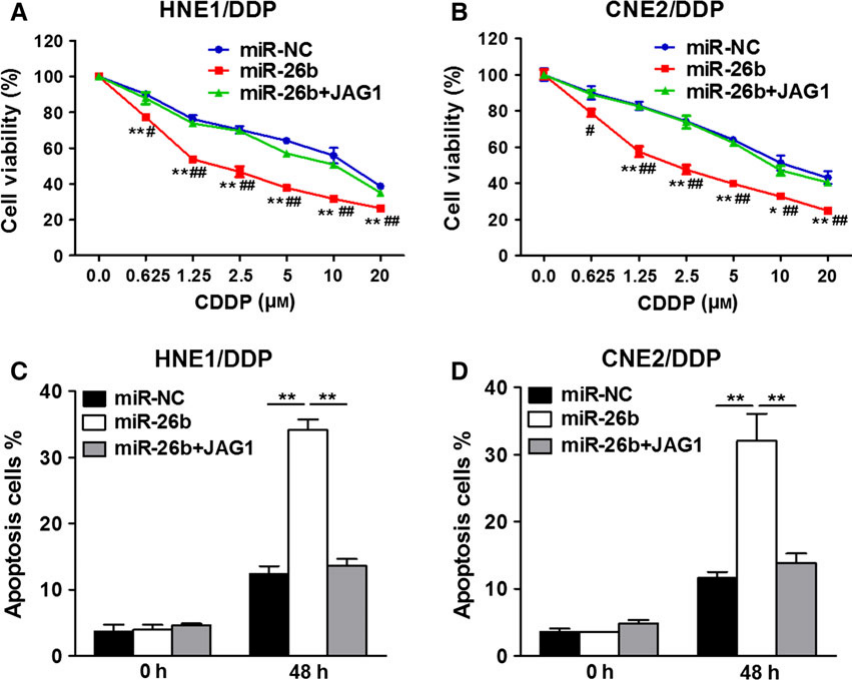
miR‐26b regulates CDDP chemosensitivity by targeting JAG1 in NPC drug‐resistant cells. (A, B) HNE1/DDP and CNE2/DDP cells stably expressing miR‐NC or miR‐26b in combination with JAG1 overexpression were treated with different concentrations of CDDP for 48 h, and analyzed by CCK‐8 assay. *P < 0.05, **P < 0.01 indicates significant difference compared to miR‐NC+vector group. #*P* < 0.05, ##*P* < 0.01 indicates significant difference compared to miR‐26b+vector group. (C, D) HNE1/DDP and CNE2/DDP cells stably expressing miR‐NC or miR‐26b in combination with JAG1 overexpression were treated with 10 μm CDDP for 0 or 48 h and cell apoptosis was analyzed by flow cytometry. **P < 0.01.

Having shown that miR‐26b inhibits JAG1 expression, we reasoned that overexpression of JAG1 could restore CDDP resistance in NPC even under miR‐26b overexpression. Indeed, as shown in Fig. 4, while miR‐26b overexpression induced a lower cell viability, cells overexpressing both miR‐26b and JAG1 demonstrated similar cell viability to cells only overexpressing miR‐NC (Fig. 4A,B). Lower apoptotic rates were also seen in cells receiving both miR‐26b and JAG1 (Fig. 4C,D). This evidence indicates that JAG1 is a target of miR‐26b and the JAG1/miR‐26b axis is indispensable in maintaining chemoresistance in NPC.

### FOXD3 regulates miR‐26b in CDDP‐resistant NPC cells

**Figure 5:**
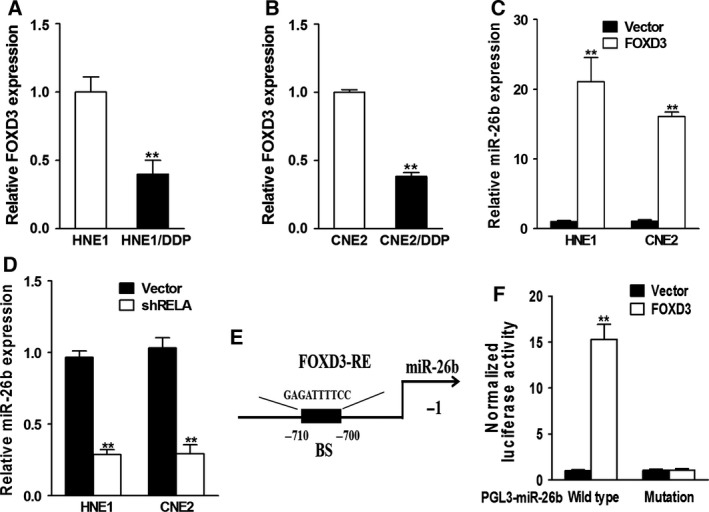
Down‐regulation of FOXD3 suppresses miR‐26b in CDDP‐resistant NPC cells. (A, B) Relative FOXD3 expression levels of paired NPC CDDP‐resistant or CDDP‐sensitive cells were analyzed by qRT‐PCR. GAPDH RNA levels were used as internal control. **P < 0.01. (C) HNE1 and CNE2 cells were transfected with Vector or FOXD3, and the relative miR‐26b expression levels were analyzed by qRT‐PCR after 24 h. **P < 0.01. (D) HNE1 and CNE2 cells were transfected with Vector or shFOXD3, and the relative miR‐26b expression level were analyzed by qRT‐PCR after 24 h. **P < 0.01. (E) Schematic diagrams show the potential FOXD3‐binding site (BS) of miR‐26b promoters. (F) Promoter luciferase reporter assay was performed on HNE1 to detect the relative luciferase activities of WT and mutant PGL3‐miR‐26b reporters. Renilla luciferase vector was used as an internal control. **P < 0.01.

Forkhead box D3 is a potential regulator of miR‐26b. To explore the relationship between FOXD3 and miR‐26b, we first characterized the expression of FOXD3 in HNE1 and CNE2 cells. It was found that FOXD3 was also down‐regulated in the CDDP‐resistant NPC (Fig. 5A,B). Ectopic expression of FOXD3 up‐regulated miR‐26b expression (Fig. 5C). Conversely, suppressing FOXD3‐RELA down‐regulated miR‐26b expression (Fig. 5D). Bioinformatic analysis indicated that FOXD3 binds to the promoter region of miR‐26b, at the position of −710 to 700 bp (Fig. 5E). This is further supported by luciferase reporter assay between FOXD3 and wild‐type miR‐26b or mutant miR‐26b promoter regions, showing that FOXD3 was positively correlated with miR‐26b (Fig. 5F).

## Discussion

Major factors that contribute to NPC carcinogenesis include genetic susceptibility, environmental factors and Epstein–Barr virus (EBV) infections. Emerging evidence indicates that the dysregulation of miRNA is also a major contributor to NPC progression. The heterogeneity of NPC determines that while some NPCs are sensitive to certain chemotherapy drugs, others may develop chemoresistance. In fact, even a small population of drug‐resistant NPC cells may eventually develop into advanced‐stage disease, rendering therapies against further progression difficult. Unfortunately, due to the lack of biomarkers for malignant NPC, a majority of NPC is diagnosed at an advanced stage. Therefore, an effective diagnostic biomarker for NPC is critical to improve the clinical outcome. Here, we demonstrated that miR‐26b was down‐regulated in CDDP‐resistant NPC cell lines. In addition, the miR‐26b down‐regulation was also demonstrated in human NPC tissue and secondary tumors. Therefore, detection of the miR‐26b down‐regulation may help identify tumors that are resistant to CDDP treatment at an early stage. The clinical management of NPC can therefore be improved by providing timely treatment, including surgery, chemotherapy, and radiation therapy to prevent disease progression and improve the clinical outcome of patients.

One approach to treat CDDP‐resistant NPC is to restore the CDDP sensitivity through miR‐26b. Here, we show that ectopic expression of miR‐26b decreased cell viability when exposed to CDDP, and increased cell apoptosis. Thus, miR‐26b could potentially act as a novel therapeutic molecule to combat cancer resistance. To date, delivery of miRNA into cancer cells has been achieved using viral and nonviral carriers 19, 20. For example, adeno‐associated virus (AAV) was used to deliver miR‐26a systemically in a mouse model of hepatocellular carcinoma 20, which showed pronounced inhibition of cancer cell proliferation, induction of tumor‐specific apoptosis, and dramatic disease in cancer progression. Combined with CDDP treatment, delivery of miR‐26b into tumors is a promising strategy for treating advanced‐stage NPC patients. This strategy is also advantageous over conventional therapeutics in that miR‐26b is well tolerated in normal tissues since miR‐26b is constitutively expressed at high levels in normal tissues.

To understand how miR‐26b regulates NPC chemoresistance, it is imperative to identify the target of miR‐26b. Previously, miR‐26b was found to target PTGS2 21, Mcl‐1 22, and pRb 23, which are key regulators of cell apoptosis. In the present study, we identified JAG1 as a novel target of miR‐26b. Elevated JAG1 expression was seen in NPC, particularly in secondary metastases. JAG1 protein is widely considered a ligand for Notch receptor. Binding between JAG1 and Notch receptor triggers the release of intracellular domain of Notch, and subsequently activates a number of oncogenes 11. Notch signaling pathway is a regulator of tumor chemoresistance, and has become a putative target of many cancer therapeutic agents 24, 25. Here, we found that miR‐26b‐binding sites exist within 3′ UTR of JAG1. Dual‐luciferase reporter assay indicated that miR‐26b inhibits the expression of JAG1. This is consistent with the fact that both miR‐26b down‐regulation and JAG1 up‐regulation are characteristics of CDDP‐resistant NPC. Interestingly, miR‐26b overexpression decreased the level of JAG1 in NPC cells, and expressing JAG1 and miR‐26b at the same time restored the chemoresistance in NPC. Therefore, the miR‐26b/JAG1 axis is essential for maintaining NPC chemoresistance. Notably, miR‐26b overexpression is not sufficient to sensitize NPC to CDDP in the presence of JAG1 overexpression. Consequently, effective treatment of CDDP‐resistant NPC should target other factors that also contribute to JAG1 overexpression.

Besides, we show that FOXD3 regulates miR‐26b expression by targeting the promoter of miR‐26b. FOXD3 is a putative tumor suppressor that affects growth, invasion, metastasis, and angiogenesis of a number of cancers, including neurobastoma 26, breast cancer 27, gastric cancer 28, and the deficiency of FOXD3 expression is linked to their malignant phenotypes. However, little is known about the role of FOXD3 in NPC. In addition, how FOXD3 exerts suppressive effects is still unclear. Previously, FOXD3 was shown to regulate in hepatocellular carcinoma growth and metastasis by targeting miR‐137 29, 30. Our study extends our knowledge on the underlying mechanism of the tumor suppressing function of FOXD3 by showing that FOXD3 also suppresses NPC by regulating miR‐26b. Moreover, we also established the link between FOXD3 and JAG1, both of which are valuable biomarkers for NPC. With respect to therapy, up‐regulating FOXD3 may also be a viable therapeutic option for NPC.

## Conclusions

Taken together, for the first time, we have demonstrated that down‐regulation of miR‐26b is associated with the chemoresistance in NPC. JAG1 is a target of miR‐26b and FOXD3 regulates miR‐26b expression. The diagnostic and therapeutic value of miR‐26b may enable accurate risk‐stratification of NPC by identifying CDDP‐resistant NPC and afford effective therapy to advanced‐stage diseases. Our findings suggest that other miRNA, such as miR‐26a, that exert similar functions to miR‐26b may also be favorable targets for NPC.

## Author contribution

LS, WY, ZZ acquired the data, analyzed and interpreted the data. LS and GS conceived, designed the project, and wrote the paper.

## Supporting information


**Fig. S1.** Establishment of miR‐26b or miR‐NC overexpressed cells.
