# Supplementary material for: Down‐regulation of miR‐26b induces cisplatin resistance in nasopharyngeal carcinoma by repressing JAG1
Source: FEBS Open Bio. 2016 Oct 24;6(12):1211–9. doi: 10.1002/2211-5463.12135 (PMC5302062; doi:10.1002/2211-5463.12135)
Supplement: Supplementary file 1 — Fig. S1. Establishment of miR‐26b or miR‐NC overexpressed cells. [file FEB4-6-1211-s001.docx]

**
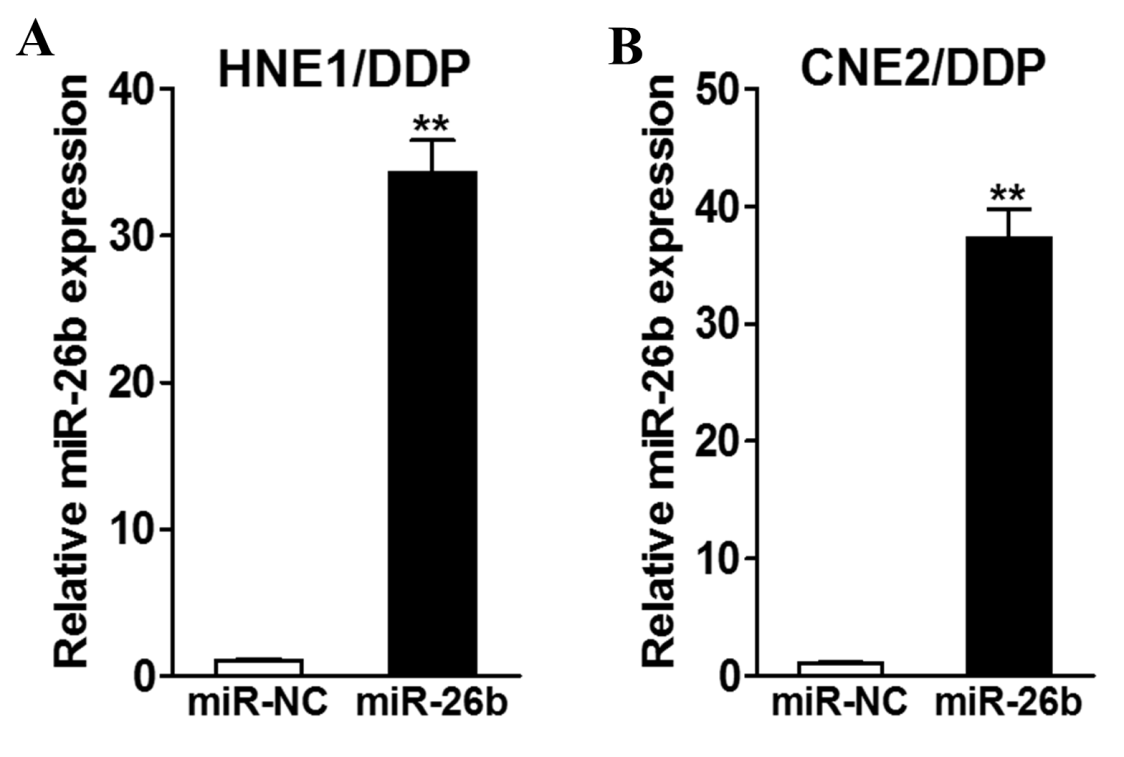
**

**Figure S1. Establishment of miR-26b or miR-NC overexpressed cells.** (A, B) The NPC cell line HNE1/DDP and CEN2/DDP were infected with miR-26b or miR-NC lentivirus to establish stable cell lines and the expression levels of miR-26b were determined by qRT-PCR and U6 levels were used as internal control, and normalized to the values of control. ** p<0.01.
